# Important components for Dutch in‐home care based on qualitative interviews with persons with dementia and informal caregivers

**DOI:** 10.1111/hex.13118

**Published:** 2020-10-07

**Authors:** Isabelle Vullings, Nanon Labrie, Joost D. Wammes, Esther W. de Bekker‐Grob, Janet MacNeil‐Vroomen

**Affiliations:** ^1^ Section Geriatrics Department of Internal Medicine Location Academic Medical Center Amsterdam University Medical Center The Netherlands; ^2^ Faculty of Sciences Athena Institute Vrije Universiteit Amsterdam The Netherlands; ^3^ Erasmus Choice Modelling Centre Erasmus University Rotterdam Rotterdam The Netherlands; ^4^ Erasmus School of Health Policy & Management Erasmus University Rotterdam Rotterdam The Netherlands

**Keywords:** accessibility of health services, dementia, health‐care economics and organizations, home‐care service, informal care, patient preference, qualitative research

## Abstract

**Background:**

Dementia care in the Netherlands is increasingly dependent on informal care and has the aim to keep persons with dementia at home for as long as possible. However, little is known about the preferences and needs of people with dementia living at home. Including people with dementia and their informal caregivers in research and policy creation could help to identify necessary forms of support, and tailor care to their personal preferences and needs.

**Objective:**

To identify important components of in‐home care for persons with dementia and their informal caregivers in the Netherlands.

**Design:**

Semi‐structured interviews across the Netherlands, between March and June 2019 using thematic analysis.

**Setting and participants:**

Persons with dementia (*n* = 5) and informal caregivers (*n* = 14) were primarily recruited through dementia care organizations. Additionally, a case manager was recruited to reflect upon the semi‐structured interviews findings.

**Results:**

Five themes concerning important care components were identified including the need for: a social network, formal care, information, emotional support and easier access to care. The complexity of the dementia care system posed a common difficulty for persons with dementia and informal caregivers.

**Conclusion:**

This study suggests that a dementia care package should be developed that includes both informal and formal care, the provision of information and emotional support, and help with access to care. The creation of this care package could help to tailor dementia care to the preferences and needs of the persons with dementia and their informal caregivers.

## 1 INTRODUCTION

The Netherlands has the highest long‐term care expenditures compared to all other member countries of the Organization for Economic Co‐operation and Development.^1^, ^2^ ^.^ It spends 3.7% of its gross domestic product on long‐term care; double the average of other countries.^1^ In 2015, the Netherlands implemented extensive reforms to their long‐term care in an attempt to control expenditure growth and provide sustainable long‐term care. The reform consisted of (a) reorienting norms, resulting in several social care services being provided by family members and local community networks instead of formalized care; (b) a shift from residential to non‐residential care, (c) decentralization of non‐residential care such as home care and (d) expenditure cuts.^3^ Moreover, approximately 600 care homes and nursing homes were closed.^3^, ^4^, ^5^

As persons with dementia make up a substantial proportion of nursing home populations,^3^ these reforms dramatically changed their care. Dementia is known as the one of the leading causes of death in older adults and the most costly disease^6^ in the Netherlands, costing 6.6 billion euros in 2015, corresponding to 7.7% of the total Dutch health‐care expenditures.^7^, ^8^ Franke et al^7^ found that since the reforms, the number of crisis situations (unplanned hospitalizations and nursing homes admissions) and the burden on informal caregivers increased. These developments imply an increased need to understand how to prevent crisis situations for persons with dementia and their informal caregivers living at home.

Despite the need for evidenced‐based policy to ensure proper health‐care services, people with dementia and informal caregivers are frequently excluded from policy creation; therefore, little is known about their personal preferences or needs. There is a plethora of in‐home support options available in the Netherlands, such as help with personal care or daycare facilities.^6^ However, it is unclear if these options are known to the target population and if the care options are aligned with, or sufficient for the preferences and needs of the people with dementia and their informal caregivers.

A previous UK‐based study by Chester et al^9^ included person with early‐stage dementia and their informal caregivers in research, identifying their personal preferences for home‐care services. The results of this study indicated that persons with early‐stage dementia preferred opportunities for social and recreational activities and support with their personal feelings and concerns.^9^ The informal caregivers appeared to prefer support with personal feelings and concerns and information on coping with dementia.^9^ Additionally, another UK study by Kampanellou et al^10^ that investigated the care preferences of informal caregivers of people with later‐stage dementia demonstrated that respite care and daily living assistance were the most preferred attributes for the informal caregivers of people with later‐stage dementia. However, as the Dutch health‐care system differs from the health‐care system in the UK, it is unclear if these findings are applicable to the Dutch dementia population.

In this study, we used semi‐structured interviews and had an in‐depth discussion with an expert, to identify important components of in‐home care for persons with dementia and their informal caregivers in the Netherlands. By including not only informal caregivers, but also persons with dementia, unique insights about their preferences and needs can be obtained. These findings can be used to build dementia care packages for the improvement of dementia care, enabling persons with dementia to stay at home for as long as possible and to avoid crisis situations such as unplanned hospitalizations or nursing home admissions. Additionally, including persons with dementia and informal caregivers in research and the development of care packages is an important component of patient‐centred care and will help patient empowerment.

## 2 METHODS

### 2.1 Study design

This qualitative study was conducted between March and June 2019 across the Netherlands. In the first phase of the study, semi‐structured interviews were held with persons with dementia and informal caregivers. Afterwards, there was an in‐depth discussion with a case manager to reflect on the findings. All interviews were held in Dutch. Based on the study protocol, the Ethics Committee (METC) of the Amsterdam University Medical Center waived the obligation for the study to undergo formal ethical approval as described in the Medical Research in Humans Act, with the reference number: W19_126#19.159. Written informed consent was obtained from the participants prior to all interviews.

### 2.2 Study participants

Persons with dementia and informal caregivers were approached. Several organizations assisted with recruitment, including Odense Huis (daycare for persons with dementia) and Alzheimer Nederland (Dutch Alzheimer Association). Participants were recruited from rural as well as urban areas in the Netherlands. Participants were contacted via telephone or email to provide further explanation about the study and to confirm their willingness to participate. Exclusion criteria consisted of (a) people who were cognitively impaired to the extent that no conversation could be held with them; (b) people who did not provide informed consent; and (c) people who were unable to speak Dutch or English. In total, 13 interviews were held, including five people with dementia and 14 informal caregivers. Four persons with dementia were interviewed together with their informal caregivers and in two interviews informal caregivers were interviewed simultaneously. The remaining participants were interviewed separately. Following these interviews, saturation was reached as no new themes or codes emerged from the data. Finally, a case manager was recruited to reflect upon the research findings of study phase one.

### 2.3 Data collection

All interviews were conducted at the participants’ homes. The interview guide consisted of broad questions about the in‐home care to identify the most important factors for the participants. Additionally, more detailed questions were included focused on the findings of previous research focusing on unmet needs of informal caregivers and persons with dementia.^9^, ^10^ Detailed information about the interview guide can be found in Table 1. The interview guide was improved through several rounds of feedback among the research team. During the interview process, the interview guide was iteratively reviewed and revised when new topics were introduced. All interviews were audio‐recorded, transcribed verbatim and translated from Dutch into English by the research team. The interviews lasted between 22 and 93 minutes.

**TABLE 1:** Topics and probing questions of the interview guide

	Topic	Example of probing question
1.	Satisfaction related to in‐home care	*Which forms of care or support did you use lately?*
		*How did you experience this care?*
2.	Help with activities of daily living	*Do you receive help with household tasks?*
		*Are you satisfied with this help?*
3.	Memory training	*Do you use tools to help you remember?*
4.	Adaptations in the home	*Were there adaptations made to your home?*
5.	Respite care/ Day care	*Do you sometimes get a break for your caregiving tasks?*
		*Do you go to a daycare facility?*
6.	Education and advice	*Do you get help or information on what dementia entails?*
		*Do you get help or information on coping with dementia?*
7.	Emotional support	*Is there somewhere you can go with you worries?*
		*Is there somewhere you can express your feelings?*
8.	Social involvement	*Do you participate in certain social activities (playing cards etc)?*

### 2.4 Data analysis

To analyse and identify relevant patterns in the data set, thematic analysis was performed in which codes and themes were derived from the data.^11^ Thematic analysis is useful when aiming to derive meaning from a data set, taking both an inductive and a deductive approach. That is, the data are analysed starting from certain (theoretically or empirically driven) assumptions, and with an open approach. The interviews were analysed and coded independently by multiple researchers (JMV, JW and IV). Subsequently, findings were compared and discussed, and a coding scheme was developed iteratively. MAXQDA 11^12^ was used for data analysis.

## 3 RESULTS

### 3.1 Participant characteristics

Table 2 presents an overview of the participants’ characteristics. In total, 20 participants were included: five persons with dementia, 14 informal caregivers and one case manager. The five key themes concerning participants’ care preferences and needs that emerged from the data consisted of: (a) importance of the social network, (b) importance of formal care, (3) need for information, (d) need for emotional support and (e) need for easier access to care. These will be discussed in detail below.

**TABLE 2:** Characteristics of the study population

NR.	Participant	Dementia stage	Living situation	IC involved	Social network involved
1.	Person with dementia	Later‐stage dementia	With partner	Yes	Family, neighbours
2.	Person with dementia	Early‐stage dementia	With partner	Yes	Family
3.	Person with dementia	Early‐stage dementia	With partner	Yes	‐
4.	Person with dementia	Early‐stage dementia	With daughter	Yes	Family
5.	Person with dementia	Middle‐stage dementia	Alone	Yes	Family, neighbours

NR.	Participant	Relation to PwD	Employment status	Multiple IC^a^	Social network involved
6.	Informal caregiver	Neighbours of PwD	Retired	No	Neighbourhood
7.	Informal caregiver	Partner of PwD	Retired	No	‐
8.	Informal caregiver	Partner of PwD	Working	Yes	Family, neighbourhood
9.	Informal caregiver	Partner of PwD	Working	Yes	Family
10.	Informal caregiver	Partner of PwD	Retired	Yes	Family
11.	Informal caregiver	Partner of PwD	Retired	Yes	Family
12.	Informal caregiver	Daughter of PwD	Working	Yes	Family
13.	Informal caregiver	Daughter of PwD	Retired	No	‐
14.	Informal caregiver	Daughter of PwD	Working	Yes	Family, neighbourhood
15.	Informal caregiver	Partner of PwD	Retired	Yes	Family
16.	Informal caregiver	Daughter of PwD	Retired	Yes	Family, neighbourhood
17.	Informal caregiver	Son in law of PwD	Retired	Yes	Family, neighbourhood
18.	Informal caregiver	Son of PwD	Working	No	‐
19.	Informal caregiver	Grandchild of PwD	Student	Yes	Family, neighbourhood

Abbreviations: IC: informal caregiver; NR.: number; PwD: person with dementia.

^a^ Multiple IC: only if multiple people provide informal care on a regular basis.

### 3.2 Importance of the social network

Informal care provided through the social network of the persons with dementia played an important role in the lives of persons with dementia and their ability to live at home. Informal caregivers helped the persons with dementia with a wide variety of tasks such as grocery shopping, finances or going to doctors’ appointments. These were often tasks that fell outside the scope of the formal caregivers, who were not allowed to help with these sorts of activities. However, as the interviewed case manager emphasized, especially for persons with dementia, it is important that there is more time to help with other sorts of daily activities.

A lot of big home‐care corporations say you (their home care staff) can only help with washing, taking medication, and bathing and then you have to go on to the next client. When especially in this patient group [persons with dementia] it is also important that you have some extra time. Participant 20 (case manager)

This vision was shared by some of the informal caregivers that indicated that they felt there was a lot of attention to the more medical side of support but that more attention should be paid to the other aspects of daily living, such as nutrition, exercise and social contact.

Exercise, nutrition, and yes actually all aspects around living. There is a lot in the area of care, but I believe that you have to find things that support people in what they can do, and I really missed that. Participant 12 (informal caregiver)

### 3.3 Importance of formal care

In addition to the satisfaction with informal care, most persons with dementia indicated that they were also pleased with the formal care provided. Both informal caregivers and persons with dementia indicated they had a lot of services available to them that they considered to be valuable and helpful to help stay at home for as long as possible. These valued services were as follows: the daycare facility, where people with dementia participated in various activities and came in contact with other people; help with daily activities, which included help with personal care, meal services, and someone that came to remind and help the person with dementia to take their medication; and finally adaptations (eg hand grips in the bathrooms) made in the home which increased the safety of the living situation.

Participants reported mixed satisfaction with case management services. Some persons with dementia and informal caregivers indicated that their case manager was very involved. These case managers provided them with information, guided them in the arrangement of care and provided a lot of attention for their personal needs and well‐being.

Yes, he [the case manager] really helps with the entire process and things that you cannot foresee. (…) The case manager is an expert and has a very important task. He can comfort you, he can arrange things if necessary, for example, placement on a waiting list. (…). He can assess the situation and is a sounding board if the care at home needs to be scaled up. Then yes, it is nice if you can consult with someone. participant 18 (informal caregiver)

Other persons with dementia and informal caregivers described their case manager as less involved and concerned with their situation. They explained that the case manager did not help them sufficiently in the arrangement of care or search for information. Some informal caregivers also indicated that they were not always sure what their case manager was supposed to help them with and that they found it hard to know if they were asking the right questions.

[About the case manager] She was a nice person but, well, she was of no use to us. Things that we asked her to help us with, well she said she would like to help but in reality, it never got done. At some point it just kind of stopped. participant 3 (person with dementia)

These differences in satisfaction with case management were acknowledged by the case manager interviewed in this study. Additionally, the case manager indicated that there might be regional differences in case management. He explained that dementia care is operated regionally and that in some regional chains, things are organized better than in others. Additionally, the case manager indicated that there are differences among health insurers regarding the importance given to dementia care and the money available for dementia care. This means that in some regions, people are offered more hours of case management than the people in other regions.

### 3.4 Need for information

Even though some participants received useful and desired information from their case manager, all informal caregivers emphasized that a lot of questions were raised by the dementia diagnosis and the process of taking care for the person with dementia. This information need can be divided into three different areas. First, persons with dementia and informal caregivers expressed a need for information about what a diagnosis with dementia entails and how they can expect the disease to evolve in the future. Notably, many participants, especially informal caregivers, indicated that they found the provision of this information, at the point of diagnosis, insufficient.

An amazing geriatrician, whom my husband really liked (…) Who examined him, very pleasant and eventually he said I am sorry you have Alzheimer’s [disease] and there is nothing more I can do for you. Well, there you are and the only thing he then said was it would be very nice if you want to participate in a study. (…) I have to say it was a disgrace. Participant 10 (informal caregiver)

Second, informal caregivers described a need for information and tips on how to cope with certain behaviours of the person with dementia. Informal caregivers indicated that they wanted to know which difficulties or aspects they should pay attention to in the home situation. Additionally, they wanted to know the best way to respond to certain dementia symptoms or behaviours of the person with dementia.

You have a lot of questions, but your parents [with dementia] have questions too (…) You are looking for practical tips, things you can expect, explanations for the behavior of your parents. How to react to them, what is wise, should you go along with it or not. participant 12 (informal caregiver)

Finally, informal caregivers and persons with dementia indicated the need for information about the care and support options available. Many participants explained that it was hard to get an overview of the available care services and that they were often unaware of certain possibilities. This was also illustrated during the interviews when some informal caregivers appeared to be unaware that they had the right to a case manager or did not even know what a case manager was. Persons with dementia and informal caregivers indicated that it would be useful if they were provided with an overview of the possibilities in terms of care and support services.

Interviewer: Ok, but did you know that case management was a possibility? Was it offered to you by, for example, the general practitioner? Respondent: No. participant 16 (informal caregiver)

### 3.5 Need for emotional support

In addition to the need for information, many participants indicated a need for emotional support. Especially the informal caregivers indicated that taking care of the person with dementia was emotionally trying and that they often felt the need to express their worries or feelings. Some informal caregivers found this emotional support in support groups with other informal caregivers. These groups gave them a space to talk about the things that they were going through. Additionally, the informal caregivers mentioned that they learned from the other informal caregivers as they discussed common issues and their solutions or coping strategies to these issues. These support groups were often offered by the daycare facilities or case managers.

I had a lot of help from the mornings here [daycare facility], the informal caregiver consultation hour, the informal caregiver support group. Where you could talk about things that you could not talk about anywhere else, things you did not want to talk about. (…) You learned from each other. We cried together, we laughed together. It meant a lot to me. participant 9 (informal caregiver)

However, not all informal caregivers had access to, or knew about, these informal caregiver support groups. This left several informal caregivers with an unfulfilled need for emotional support. These participants indicated that they would have liked to have someone to share their worries or emotions with.

I think it would be a good plan that when something like that is over [taking care of a person with dementia], but also when it is still happening, that you [as informal caregiver] have help. Just someone to talk to sometimes. I think I really need that sometimes. participant 6 (informal caregiver)

### 3.6 Need for easier access to care

The persons with dementia and informal caregivers also experienced difficulties with access to care. First, it is important to note that if people are unaware of the care services available to them, this means they will not be able to access these care services. Furthermore, both persons with dementia and informal caregivers expressed that they found the dementia care system complicated and fragmented. For example, how care was delivered and reimbursed by different institutions or legal acts was confusing to people. Some persons with dementia and informal caregivers who reported these difficulties with the access to care did not have a case manager or were dissatisfied with their case manager. They explained that they had to arrange care themselves, which they found complicated and time‐consuming. The participants indicated that it was hard to get on overview of where they needed to be or who they needed to contact for which services or form of support.

In the end, you have to look into it yourself, and yes then you get telephone numbers and addresses and you have to get started with that. Well, yes you need to have a lot to offer in order to get that done. Cause I can imagine that many people do not know where they need to search for these things. That they are overwhelmed. participant 7, (informal caregiver)

The Dutch legal framework for long‐term care posed an additional problem for the accessibility of care for people with dementia living at home. These problems were caused by the Long‐term Care Act (WLZ). The case manager in this study explained that when a person with dementia wants to be on the waiting list for a long‐term care facility, they need to apply for a long‐term care indication. When a long‐term care indication is there, the indication for the Social Support Act (WMO) ends and care will no longer be financed through the WMO. This means that all care received by the persons living at home, waiting to be placed in a long‐term care facility, will be financed through the WLZ. However, the budget of the WLZ is exponentially lower than the budget from the WMO and the Health Insurance act (ZVW), which means that either less care can be offered, or people need to start paying more out of pocket. As the out‐of‐pocket costs are quite expensive, many people may not be able to afford the services based on their fixed retirement incomes. The case manager described this as worrisome, since people that are waiting to be moved to a long‐term care facility are often vulnerable and in need of extensive care and support.

The moment you get a WLZ indication, your WMO indication stops and then you have to start paying your homecare, visits to the daycare facility, and domestic help through the WLZ indication. However, you have a weekly budget and that is lower with the WLZ indication than it was with the WMO indication. participant 20 (case manager)

## 4 DISCUSSION

### 4.1 Key findings

This study shows that Dutch persons with dementia as well as their informal caregivers are generally pleased with the formal and informal care that they received. This is consistent with a previous study in which Dutch informal caregivers had a positive judgement of the professional dementia care and support, throughout different regions in the Netherlands.^13^ Both persons with dementia and informal caregivers in this study commonly used and valued services such as the daycare facilities, help with daily activities from home‐care organizations and adaptations in the home to increase safety. Additionally, the participants in this study, especially the informal caregivers, indicated to have a need for emotional support and information. Even though some informal caregivers reported that they found and appreciated certain forms of emotional support, other informal caregivers reported to have unfulfilled support and information needs and were often unaware of the services available to them.

This need for information and emotional support, especially for the informal caregivers, has been identified in previous Dutch studies.^14^, ^15^ Additionally, a previous UK study by Chester et al^9^ found that the most preferred home support options for caretakers of people with early‐stage dementia included ‘support with personal feelings and concerns – provided by a trained counselor at home’ and ‘information on coping with dementia – provided by an experienced worker at home’. Furthermore, a study of Kampanellou et al^10^ indicated that for the informal caregivers of people with later‐stage dementia, respite care was the most preferred attribute for in‐home support. The informal caregivers in this study did not talk about a need for respite care. However, almost all participants explained that the person with dementia went to a daycare facility several times a week. For the informal caregiver, this meant that they were temporarily released from their caregiving tasks, which could therefore be seen as a form of respite care.

Furthermore, this study shows that both persons with dementia and informal caregivers deem dementia care complex and express the need for guidance and help with the access to care. Some participants found this help and guidance in their case manager, whereas others were very dissatisfied with their case manager. These differences in satisfaction might be related to the fact that financing of dementia care is dependent on the policy of the municipalities, which puts the continuity of case management at risk.^16^ Additionally, there are regional differences in the waiting times for case management, in some regions there are no waiting lists, while in other regions people have to wait months to get a case manager.^6^ Furthermore, informal caregivers indicated that they did not always know what the case managers’ tasks were and what they could expect from their case manager. This made it harder for the informal caregivers to know when they could turn to their case manager for advice or guidance.^16^ Finally, there might be some individual differences between case managers in the way they realize their position. This might have to do with the differences in the position of the case manager in the care network, as well as case managers’ background.^17^ For example, case managers with a background in psychology might give more importance to the emotional well‐being of the persons with dementia and informal caregivers, while case managers that do not have this background focus less on psychological aspects.

Finally, this study shows that there are some difficulties for persons with dementia who are on waiting lists for long‐term care. Although these people are still at home, they are no longer applicable for the same services as provided by the social support act for community‐dwelling older adults (WMO) but instead services are payed and provided through the Long‐term Care Act (WLZ). This transition comes with a higher financial burden, which means that people that are living at home, waiting to be placed in a nursing home and often in need of a great amount of care, need to start paying exponentially more out of pocket. Francke et al^6^ found persons with dementia in this transition phase, lost on average, five hours of care per week. It is unclear if this is the time when more persons with dementia have crisis situations. Regardless, we recommend that the WLZ law only apply once a person is actually residing in long‐term care to avoid crisis situations.

### 4.2 Strengths and limitations

To the best of our knowledge, this study is the first interview study in the Netherlands to include persons with dementia and give them the opportunity to voice their opinions about the in‐home care that they receive. This study shows that including people with dementia in research can lead to meaningful and unique insight into their personal preferences and needs, and the improvement points of care. This study can therefore be used as an example to demonstrate that, despite their cognitive impairments, people with dementia are very much capable of expressing themselves and to articulate their thoughts and opinions about the care that they receive.

There are certain limitations to this study. First, the recruited persons with dementia in this study all lived with a partner or relative, and all had an informal caregiver. Since informal care plays a crucial role in dementia care in the Netherlands,^18^ it is only reasonable to assume that persons with dementia without an informal caregiver have different care preferences and needs than those who do have an informal caregiver. Additionally, most of the recruited persons with dementia had early‐stage dementia. When dementia advances, people often become more dependent and demanding in terms of formal and informal care.^6^, ^7^, ^8^, ^9^, ^10^, ^11^, ^12^, ^13^, ^14^, ^15^, ^16^, ^17^, ^18^, ^19^ However, there were informal caregivers included who had their loved ones currently institutionalized or had recently died. In these cases, the informal caregivers provided recent retrospective information on the care needs for later‐stage dementia.

### 4.3 Implications of the study

This study helps to tailor the dementia care to the reported preferences and needs of the persons with dementia and their informal caregivers. On the individual level, this could help to improve the quality of life of persons with dementia and informal caregivers and improve their living situation at home. On a societal level, this could help to maintain a safe sustainable living situation at home for persons with dementia and informal caregivers, which could help to save costs due to crisis situations such as acute hospitalizations or temporary nursing home admissions.

Based on the findings of this study, some aspects of dementia care that could be further improved were identified, such as the provision of information and emotional support. Information about the available care services should be increased, and the access to these care services should be simplified. The case manager should play an important role in these objectives since they are the central person when it comes to care for people with dementia living at home and their informal caregivers. Additionally, the financial problems with the transition of care from the WMO to the WLZ should be tackled, and the financing of care through the WLZ should start when the person with dementia actually moves to the long‐term care facility. Further research is needed to identify the care preferences and unmet needs of persons with dementia that do not have an informal caregiver. Additionally, future studies should use quantitative approaches, such as surveys to validate the results of this study or discrete choice experiments to determine the relative importance of the five aspects for persons with dementia and informal caregivers. These studies could help to efficiently and effectively improve the current dementia care system for persons with dementia and informal caregivers.

To conclude, in this interview study, the important components of in‐home care for persons with dementia and their informal caregivers in the Netherlands were identified. These important components included both informal and formal care, the provision of information and emotional support, and help with access to care. Creating one care package, with these identified components, would help elucidate what services are available for persons with dementia and informal caregivers and therefore make them more accessible. Moreover, including these components in dementia care packages would help to improve the dementia care in a way that it enables persons with dementia and their informal caregivers to live at home in a safe and responsible manner.

All aspects of living are important. There is a lot of support in the area of patient care, however, I believe you also need to support people in what it is they can do. You just need to look at the entire day and see what is needed in order to live it in good quality. participant 12 (informal caregiver)

## CONFLICT OF INTEREST

There was no conflict of interest for any of the authors involved in this article.

## Data Availability

The data that support the findings of this study are available on request from the corresponding author. The data are not publicly available due to privacy or ethical restrictions.

## References

[1] OECD . Health at a Glance 2017: OECD Indicators. Paris: OECD; 2017.

[2] Colombo F , Llena‐Nozal A , Mercier J , Tjadens F . Help Wanted?: Providing and Paying for Long‐Term Care. Paris: OECD Publishing; 2011.

[3] Maarse JA , Jeurissen PP . The policy and politics of the 2015 long‐term care reform in the Netherlands. Health Policy. 2015;120:241‐245. 10.1016/j.healthpol.2016.01.014 26872702

[4] Verbeek‐Oudijk D , Campen CV . Ouderen in verpleeghuizen en verzorgingshuizen Landelijk overzicht van hun leefsituatie in 2015/ ’16. In: Planbureau SeC, editor. Den Haag; 2017.

[5] Aedes‐Actiz Kenniscentrum Wonen‐Zorg. Onderzoek naar sluiting verzorgingshuizen. Utrecht.

[6] Dutch Central Bureau of Statistics. Dementia. The Hague.

[7] Francke A , van der Heide I , De Bruin S Gijsen R , Poos R , Veerbeek M , et al. Een samenhangend beeld van dementie en dementiezorg: kerncijfers, behoeften, aanbod en impact. Themarapportage van de Staat van Volksgezondheid en Zorg. www.nivel.nl: Nivel, 2018.

[8] Alzheimer Nederland. Factsheet cijfers en feiten over dementie. 2019 Retrieved from https://www.alzheimernederland.nl/factsheet‐cijfers‐en‐feiten‐over‐dementie

[9] Chester H , Clarkson P , Davies L , et al. People with dementia and carer preferences for home support services in early‐stage dementia. Aging Ment Health. 2018;22:270‐279. 27849124 10.1080/13607863.2016.1247424

[10] Kampanellou E , Chester H , Davies L , et al. Carer preferences for home support services in later stage dementia. Aging Ment Health. 2019;23:60‐68. 29090948 10.1080/13607863.2017.1394441

[11] Braun V , Clarke V . Using thematic analysis in psychology. Qual Res Psychol. 2006;3:2:77‐101. 10.1191/1478088706qp063oa

[12] Software V . MAXQDA, 11 edn Berlin: VERBI Software; 2014. p. Computer Software.

[13] Jansen D , Werkman W , Francke AL . Dementiemonitor Mantelzorg 2016. Utrecht: NIVEL; 2016.

[14] Lauriks S , Reinersmann A , Van der Roest HG , et al. Review of ICT‐based services for identified unmet needs in people with dementia. Ageing Res Rev. 2007;6:223‐246. 17869590 10.1016/j.arr.2007.07.002

[15] Peeters JM , Van Beek AP , Meerveld JH , Spreeuwenberg PM , Francke AL . Informal caregivers of persons with dementia, their use of and needs for specific professional support: a survey of the National Dementia Programme. BMC Nurs. 2010;9:9. 20529271 10.1186/1472-6955-9-9 PMC2901350

[16] Peeters JM , De Lange J , Van Asch I , et al. Landelijke evaluatie van casemanagement dementie Nivel Trimbos‐instituut. 2012:1–135. https://nivel.nl/sites/default/files/bestanden/Rapport-casemanagement-dementie.pdf

[17] Minkman MM , Ligthart SA , Huijsman R . Integrated dementia care in The Netherlands: a multiple case study of case management programmes. Health Soc Care Community. 2009;17:485‐494. 19694030 10.1111/j.1365-2524.2009.00850.x

[18] Lethin C , Leino‐Kilpi H , Bleijlevens MH , et al. Predicting caregiver burden in informal caregivers caring for persons with dementia living at home–A follow‐up cohort study. Dementia. 2018;1471301218782502. 10.1177/1471301218782502 29929383

[19] Huang HL , Shyu YIL , Chen MC , et al. Family caregivers’ role implementation at different stages of dementia. Clin Interv Aging. 2015;10:135. 25584022 10.2147/CIA.S60574 PMC4289485

